# Molecular pathway of anticancer effect of next-generation HSP90 inhibitors XL-888 and Debio0932 in neuroblastoma cell line

**DOI:** 10.1007/s12032-024-02428-z

**Published:** 2024-07-03

**Authors:** Özlem Kaplan, Nazan Gökşen Tosun

**Affiliations:** 1 Department of Genetics and Bioengineering, Rafet Kayış Faculty of Engineering, Alanya Alaaddin Keykubat University, Antalya, Türkiye; 2 Department of Medical Services and Techniques, Tokat Gaziosmanpaşa University, Tokat Vocational School of Health Services, Tokat, Türkiye

**Keywords:** HSP90 inhibition, Neuroblastoma, XL-888, Debio0932, Cancer

## Abstract

Neuroblastoma is a common nervous system tumor in childhood, and current treatments are not adequate. HSP90 is a molecular chaperone protein that plays a critical role in the regulation of cancer-related proteins. HSP90 inhibition may exert anticancer effects by targeting cancer-related processes such as tumor growth, cell proliferation, metastasis, and apoptosis. Therefore, HSP90 inhibition is a promising strategy in the treatment of various types of cancer, and the development of next-generation inhibitors could potentially lead to more effective and safer treatments. XL-888 and Debio0932 is a next-generation HSP90 inhibitor and can inhibit the correct folding and stabilization of client proteins that cancer-associated HSP90 helps to fold correctly. In this study, we aimed to investigate the comprehensive molecular pathways of the anticancer activity of XL-888 and Debio0932 in human neuroblastoma cells SH-SY5Y. The cytotoxic effects of XL-888 and Debio0932 on the neuroblastoma cell line SH-SY5Y cells were evaluated by MTT assay. Then, the effect of these HSP90 inhibitors on the expression of important genes in cancer was revealed by Quantitative Real Time Polymerase Chain Reaction (qRT-PCR) method. The qRT-PCR data were evaluated using Kyoto Encyclopedia of Genes and Genomes (KEGG) and Gene Ontology (GO) biological process tools. Finally, the effect of HSP90 inhibitors on HSP27, HSP70 and HSP90 protein expression was investigated by Western blotting analysis. The results revealed that XL-888 and Debio0932 had a role in regulating many cancer-related pathways such as migration, invasion, metastasis, angiogenesis, and apoptosis in SH-SY5Y cells. In conclusion, it shows that HSP90 inhibitors can be considered as a promising candidate in the treatment of neuroblastoma and resistance to chemotherapy.

## Introduction

Neuroblastoma is a pediatric nervous system tumor originating from neural crest elements [1]. Neuroblastoma is the third most common solid tumor of infancy and childhood and is responsible for 15% of all pediatric cancer deaths worldwide [2]. When diagnosed with neuroblastoma, there is an aggressive scenario in which approximately 50% of patients have metastases to the bone, bone marrow and lymph nodes. Despite all recent advances, therapeutic interventions in neuroblastoma are still not sufficient. [3]. Therefore, it is important to develop new targeted treatments in cases of neuroblastoma.

Heat shock protein 90 (HSP90) has become a target in anticancer therapy due to its important roles in cancer development, especially in adhesion, angiogenesis, invasion, cell proliferation, metastasis, migration, and apoptosis [4]. Due to the presence of many HSP90 client proteins, HSP90 inhibition targets multiple signaling pathways simultaneously and may exert multimodal therapeutic effect. Many chemically diverse HSP90 inhibitors have been shown in various studies to exhibit anticancer activity [5–8]. XL-888 is an oral next-generation HSP90 inhibitor that significantly limits HSP90 activity without inhibiting other kinases [9]. XL-888 has been shown to be effective in melanoma, liver, and advanced pancreatic/colorectal cancer [10, 11]. Debio-0932 is a new generation synthetic Hsp90 inhibitor whose anticancer activities in lymphoma, solid tumors, non-small cell lung cancer and kidney cancer are being investigated. Debio-0932 has superior properties such as high tumor selectivity, blood–brain barrier penetration, high oral bioavailability [12, 13]. Despite all these promising features, there are not yet sufficient studies on the biological significance of the new generation HSP90 inhibitors XL-888 and Debio0932 in neuroblastoma.

In this study, we aimed to evaluate the therapeutic potential of both HSP90 inhibitors, XL-888 and Debio0932, in neuroblastoma by examining their effects on the expression of important cancer-related genes by a qRT-PCR analysis. For the first time, the molecular pathway of the effect of new generation HSP90 inhibitors on the neuroblastoma cell line SH-SY5Y cells was examined in detail. The effects of two HSP90 inhibitors on these cells were comprehensively evaluated.

## Materials and methods

### Materials

XL-888 and Debio0932 were purchased from AdooQ Bioscience LLC, Irvine, CA, US. SYBR green master mix (330500) and Human Cancer Pathway Finder™ PCR array (PAHS-033Z) was obtained from Qiagen. Favorgen Biotech Corp. provided the total RNA isolation kit. Bio-Rad supplied the cDNA synthesis kits and the enhanced chemiluminescence (ECL) substrate kit (1705060). SH-SY5Y cell line was purchased from ATCC (American Type Culture Collection). The 3-(4,5-dimethylthiazol-2-yl)-2,5-diphenyltetrazolium bromide (MTT) and Polyvinylidene difluoride (PVDF) membrane (741260) were provided by Macherey–Nagel. The BCA protein assay kit and RIPA lysis buffer were obtained from Serva. Abcam supplied the following antibodies: Anti-HSP27 (ab5579), anti-HSP70 (ab79852), anti-HSP90 (ab2928), and goat anti-rabbit IgG H&L (HRP) (ab205718). Dulbecco’s Modified Eagle’s medium (DMEM) High Glucose, penicillin–streptomycin solution, phosphate buffer saline (PBS), fetal bovine serum (FBS), trypsin–EDTA and L-glutamine were obtained from Biological Industries.

### Cell cytotoxicity assay

The cytotoxic effects of XL-888 and Debio0932 on neuroblastoma cell line SH-SY5Y was determined by MTT test. SH-SY5Y cells were seeded in 96-well culture plates (5 × 10^4^ cells per well) and treated with XL-888 and Debio0932 for 24 h and 48 h at concentrations ranging from 100 to 1.56 nM. Following the incubation, after the medium was removed, MTT solution (5 mg/mL) was added to each well and incubated at 37 °C for 3 h. After removing MTT, the formazan product produced by living cells, it was dissolved with 100 µL dimethyl sulfoxide (DMSO). The percentage of the cancer cell viability was calculated by measuring the absorbance values at 570 nm with a spectrophotometric microplate reader [14].

### qRT-PCR analysis

Human Cancer Pathway Finder™ PCR array was used to analyze the effect of XL-888 and Debio0932 on genes important in molecular pathways in cancer. In this assay, SH-SY5Y cells were seeded in a 25 cm^2^ cell culture flask and cells were exposed to XL-888 and Debio0932 separately for 48 h. Total RNA was isolated using a total RNA isolation kit, and these RNAs were synthesized into cDNA using a commercial cDNA synthesis kit. The qRT-PCR experiment was performed using SYBR green master mix on the BioRad CFX96™ instrument. The reaction mixture included 1 µL cDNA, 1 µL primer mix (10 mM stock), 11.5 µL SYBR Green qPCR master mix, and 10.5 µL RNase-free water. The thermal cycling conditions consisted of an initial denaturation at 95 °C for 10 min, followed by 40 cycles of denaturation at 95 °C for 15 s and annealing/extension at 60 °C for 60 s. Gene expression levels were analyzed using the 2^−ΔΔCt^ method [15]. The primers used for the qRT-PCR analyses are listed in Table 1. The genes beta-actin (ACTB), beta-2-microglobulin (B2M), and glyceraldehyde-3-phosphate dehydrogenase (GAPDH), were used for normalization in the analyses.

**Table 1:** Gene symbols and descriptions in the qRT-PCR analysis

Symbol	Description
ACLY	ATP citrate lyase
ACSL4	Acyl-CoA synthetase long-chain family member 4
ADM	Adrenomedullin
ANGPT1	Angiopoietin 1
ANGPT2	Angiopoietin 2
APAF1	Apoptotic peptidase activating factor 1
ARNT	Aryl hydrocarbon receptor nuclear translocator
ATP5A1	ATP synthase, H + transporting, mitochondrial F1 complex, alpha subunit 1, cardiac muscle
AURKA	Aurora kinase A
BCL2L11	BCL2-like 11 (apoptosis facilitator)
BIRC3	Baculoviral IAP repeat containing 3
BMI1	BMI1 polycomb ring finger oncogene
CA9	Carbonic anhydrase IX
CASP2	Caspase 2, apoptosis-related cysteine peptidase
CASP7	Caspase 7, apoptosis-related cysteine peptidase
CASP9	Caspase 9, apoptosis-related cysteine peptidase
CCL2	Chemokine (C–C motif) ligand 2
CCND2	Cyclin D2
CCND3	Cyclin D3
CDC20	Cell division cycle 20 homolog (S. cerevisiae)
CDH2	Cadherin 2, type 1, N-cadherin (neuronal)
CFLAR	CASP8 and FADD-like apoptosis regulator
COX5A	Cytochrome c oxidase subunit Va
CPT2	Carnitine palmitoyltransferase 2
DDB2	Damage-specific DNA binding protein 2, 48 kDa
DDIT3	DNA-damage-inducible transcript 3
DKC1	Dyskeratosis congenita 1, dyskerin
DSP	Desmoplakin
E2F4	E2F transcription factor 4, p107/p130-binding
EPO	Erythropoietin
ERCC3	Excision repair cross-complementing rodent repair deficiency, complementation group 3 (xeroderma pigmentosum group B complementing)
ERCC5	Excision repair cross-complementing rodent repair deficiency, complementation group 5
ETS2	V-Ets erythroblastosis virus E26 oncogene homolog 2 (avian)
FASLG	Fas ligand (TNF superfamily, member 6)
FGF2	Fibroblast growth factor 2 (basic)
FLT1	Fms-related tyrosine kinase 1 (vascular endothelial growth factor/vascular permeability factor receptor)
FOXC2	Forkhead box C2 (MFH-1, mesenchyme forkhead 1)
G6PD	Glucose-6-phosphate dehydrogenase
GADD45G	Growth arrest and DNA-damage-inducible, gamma
GPD2	Glycerol-3-phosphate dehydrogenase 2 (mitochondrial)
GSC	Goosecoid homeobox
HMOX1	Heme oxygenase (decycling) 1
IGFBP3	Insulin-like growth factor binding protein 3
IGFBP5	Insulin-like growth factor binding protein 5
IGFBP7	Insulin-like growth factor binding protein 7
KDR	Kinase insert domain receptor (a type III receptor tyrosine kinase)
KRT14	Keratin 14
LDHA	Lactate dehydrogenase A
LIG4	Ligase IV, DNA, ATP-dependent
LPL	Lipoprotein lipase
MAP2K1	Mitogen-activated protein kinase kinase 1
MAP2K3	Mitogen-activated protein kinase kinase 3
MAPK14	Mitogen-activated protein kinase 14
MCM2	Minichromosome maintenance complex component 2
MKI67	Antigen identified by monoclonal antibody Ki-67
NOL3	Nucleolar protein 3 (apoptosis repressor with CARD domain)
OCLN	Occludin
PFKL	Phosphofructokinase, liver
PGF	Placental growth factor
PINX1	PIN2/TERF1 interacting, telomerase inhibitor 1
POLB	Polymerase (DNA directed), beta
PPP1R15A	Protein phosphatase 1, regulatory (inhibitor) subunit 15A
SERPINB2	Serpin peptidase inhibitor, clade B (ovalbumin), member 2
SERPINF1	Serpin peptidase inhibitor, clade F (alpha-2 antiplasmin, pigment epithelium derived factor), member 1
SKP2	S-phase kinase-associated protein 2 (p45)
SLC2A1	Solute carrier family 2 (facilitated glucose transporter), member 1
SNAI1	Snail homolog 1 (Drosophila)
SNAI2	Snail homolog 2 (Drosophila)
SNAI3	Snail homolog 3 (Drosophila)
SOD1	Superoxide dismutase 1, soluble
SOX10	SRY (sex determining region Y)-box 10
STMN1	Stathmin 1
TBX2	T-box 2
TEK	TEK tyrosine kinase, endothelial
TEP1	Telomerase-associated protein 1
TERF1	Telomeric repeat binding factor (NIMA-interacting) 1
TERF2IP	Telomeric repeat binding factor 2, interacting protein
TINF2	TERF1 (TRF1)-interacting nuclear factor 2
TNKS	Tankyrase, TRF1-interacting ankyrin-related ADP-ribose polymerase
TNKS2	Tankyrase, TRF1-interacting ankyrin-related ADP-ribose polymerase 2
UQCRFS1	Ubiquinol-cytochrome c reductase, Rieske iron-sulfur polypeptide 1
VEGFC	Vascular endothelial growth factor C
WEE1	WEE1 homolog (S. pombe)
XIAP	X-linked inhibitor of apoptosis
ACTB	Actin, beta
B2M	Beta-2-microglobulin
GAPDH	Glyceraldehyde-3-phosphate dehydrogenase
HPRT1	Hypoxanthine phosphoribosyltransferase 1
RPLP0	Ribosomal protein, large, P0
HGDC	Human Genomic DNA Contamination
RTC	Reverse Transcription Control
RTC	Reverse Transcription Control
RTC	Reverse Transcription Control
PPC	Positive PCR Control
PPC	Positive PCR Control
PPC	Positive PCR Control

### Western blotting

Heat shock proteins Hsp27, Hsp70, Hsp90 were detected in SH-SY5Y cells exposed to XL-888 and Debio0932 at IC_50_ doses for 48 h. The change in protein expression levels was investigated using the Western blotting technique. Western blotting was performed as detailed previously [16]. Total protein isolation was performed with RIPA buffer and total protein concentration was determined using the BCA protein assay kit. Subsequently, proteins (30 μg/well) were separated through a 12% SDS-PAGE gel and transferred to PVDF membranes. Following this, the membranes underwent blocking with 5% skim milk powder in TBST for 1 h. Primary antibodies including anti-HSP27 (1:1000), anti-HSP70 (1:500), anti-HSP90 (1:500) were incubated overnight at 4 °C. After washing with TBST, membranes were incubated with a secondary antibody (goat anti-rabbit IgG H&L (HRP)) (1:10,000) for 1 h at room temperature. The anti-GAPDH (1:5000) primary antibody was used for normalization. Protein bands were visualized using ChemiDoc™ imaging equipment (Bio-Rad) and an ECL substrate. ImageLab 6.1 software was used to analyze protein expression levels.

### Pathway analysis

Genes whose expressions changed in SH-SY5Y cells were detected by qRT-PCR analysis, and the correlation between these genes and cancer pathways was determined using the Enrichr web tool (https://maayanlab.cloud/Enrichr/). Kyoto Encyclopedia of Genes and Genomes (KEGG) and Gene Ontology (GO) biological process analyzes were performed using the Enrich web tool. The performance of the analysis was assessed by *p* value and *q* value.

### Statistical analysis

GraphPad Prism 8.0 software was used to perform a two-way ANOVA test with Sidak and Dunnett tests. Statistical significance was defined as probability values of *p* < 0.05. In pathway analysis using the Enrichr web tool, the *q* value is an adjusted *p* value determined using the Benjamini–Hochberg technique for analysis correction.

## Results and discussion

### Cell cytotoxicity assay

**Fig. 1:**
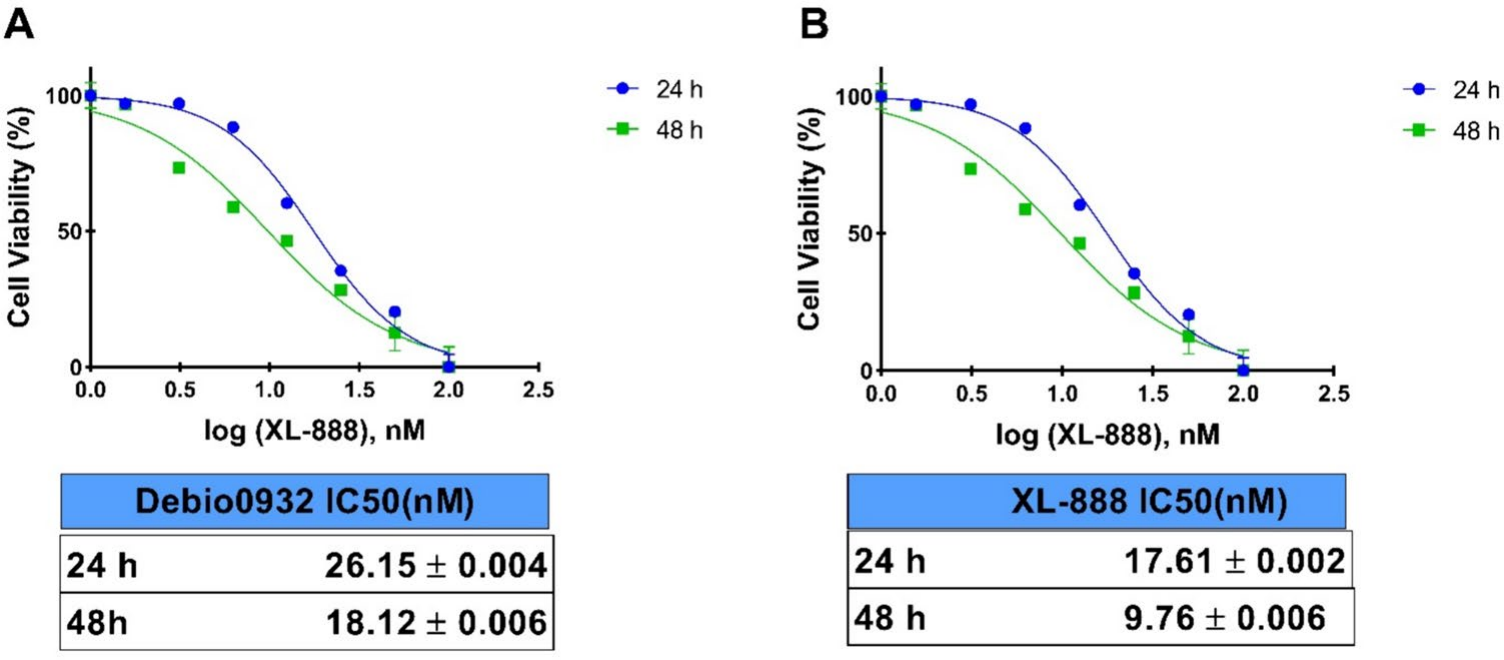
Debio0932 (**A**) and XL-888 (**B**) cytotoxicity and IC_50_ values in SH-SY5Y cells after 24 h and 48 h

The cytotoxic effect of XL-888 and Debio0932 HSP90 inhibitor on SH-SY5Y cell lines was revealed by MTT test. SH-SY5Y cells were exposed to various concentrations of Hsp90 inhibitors for 24 h and 48 h. There was a dose- and time-dependent decrease in SH-SY5Y cell viability against HSP90 inhibitors. IC_50_ values in SH-SY5Y cells exposed to XL-888 for 24 h were 17.61 nM and for 48 h were 9.76 nM. In SH-SY5Y cells treated with Debio0932, IC_50_ values were 26.15 nM and 18.12 nM for 24 h and 48 h, respectively (Fig. 1). Hanna et al. reported that the HSP90 inhibitor 17-AAG exerted a cytotoxic effect on IMR-32 and SK-N-SH human neuroblastoma cells by significantly increasing the rate of cellular apoptosis [17].

### qRT-PCR and pathway analysis

**Fig. 2:**
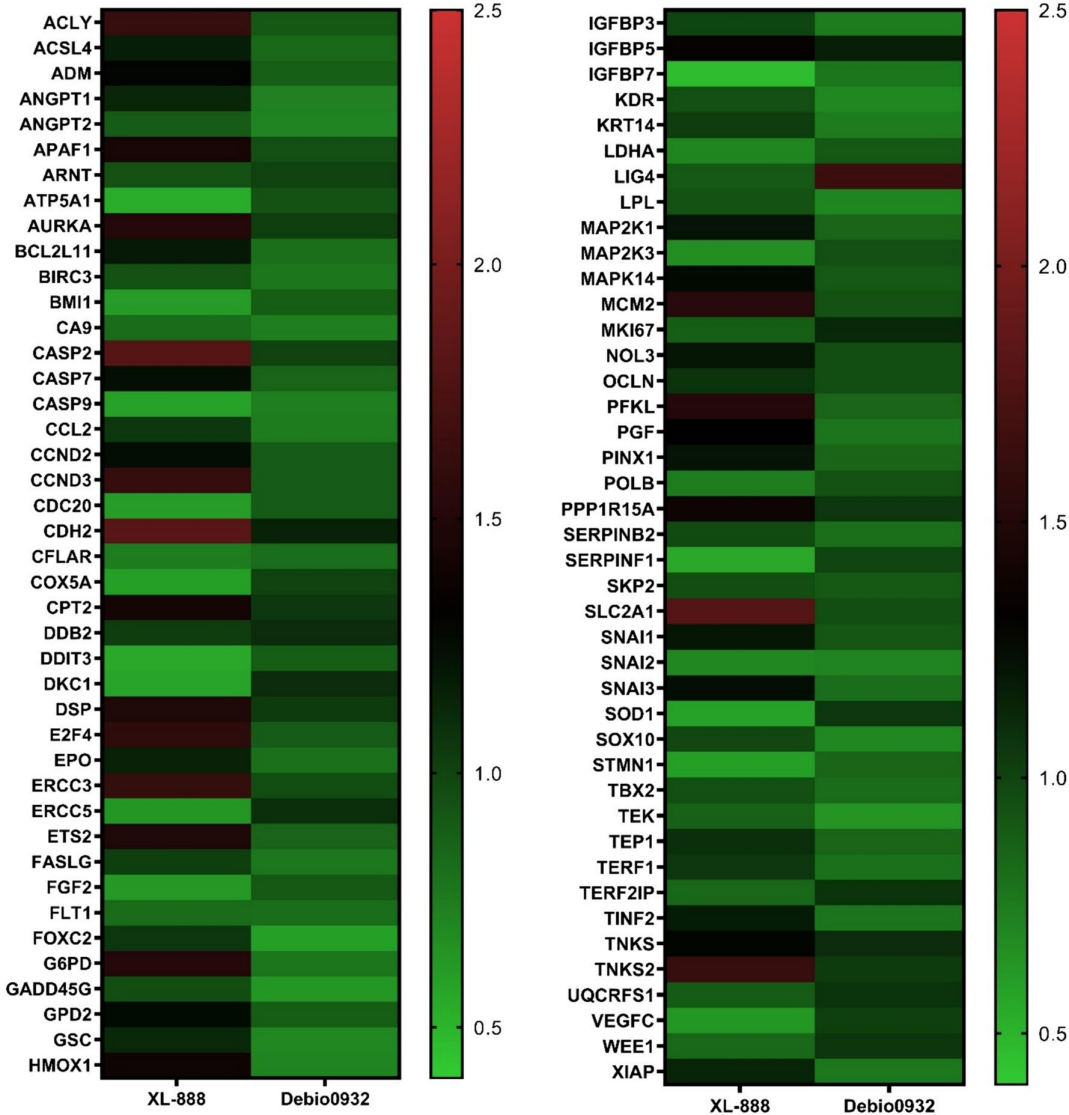
Heatmap of gene expression profiles of SH-SY5Y cells treated with XL-888 and Debio0932

A cancer pathway finder PCR array was used to determine the expression levels of cancer-associated genes in neuroblastoma cells exposed to Hsp90 inhibitors. The cells not exposed to Hsp90 inhibitors were considered as control cells, and SH-SY5Y cells were treated with the determined IC_50_ dose of Hsp90 inhibitors for 48 h. Subsequently, total RNA was isolated from these cells and qRT-PCR experiments were conducted. A list of genes whose expression was examined in the qRT-PCR analyses is presented in Table 1. The qRT-PCR analysis results showed changes in many genes involved in cancer formation and progression. Changes in gene expression in SH-SY5Y cells following exposure to Hsp90 inhibitors were depicted as a heat map in Fig. 2. Results regarding gene expression changes revealed that in SH-SY5Y cells exposed to XL-888, the genes ATP5A1, BMI1, CASP9, CDC20, COX5A, DDIT3, DKC1, ERCC5, FGF2, IGFBP7, MAP2K3, SERPINF1, SNAI2, SOD1, STMN1, and VEGFC were downregulated, while the genes ACLY, APAF1, AURKA, CASP2, CCND3, CDH2, CPT2, DSP, E2F4, ERCC3, ETS2, G6PD, HMOX1, MCM2, PFKL, PPP1R15A, SLC2A1, and TNKS2 were upregulated. In these cells, Debio0932 decreased the expression of the genes ANGPT1, ANGPT2, CA9, CASP9, FOXC2, GADD45G, GSC, HMOX1, KDR, LPL, SNAI2, SOX10, and TEK, while it increased the expression of the LIG4 gene.

The administration of HSP90 inhibitors XL-888 and Debio0932 to neuroblastoma cells resulted in a decrease in the expression of angiogenesis-related genes, including ANGPT1, ANGPT2, FGF2, KDR, TEK, VEGFC, and SERPINF1 [18]. While XL-888 led to a decrease in the expression of FGF2, SERPINF1 and VEGFC genes, Debio0932 triggered a reduction in the expression of ANGPT1, ANGPT2, KDR, and TEK genes. Our findings indicate that HSP90 inhibition with XL-888 and Debio0932 reduces the expression of angiogenesis-related genes in neuroblastoma cells and has great potential for inhibiting angiogenesis. Similarly, Zhang and colleagues have demonstrated that AT-533, an Hsp90 inhibitor, suppresses breast cancer growth and angiogenesis by inhibiting the HIF-1α/VEGF/VEGFR-2 signaling pathway [19].

XL-888 and Debio0932 resulted in alterations in the expression of apoptosis-related genes APAF1, CASP2, and CASP9 in SH-SY5Y cells. Administration of XL-888 in SH-SY5Y cells resulted in a decrease in CASP9 expression while increasing APAF1 and CASP2 gene expression. While Debio0932 showed no notable impact on APAF1 and CASP2 expression, it elicited a decrease in CASP9 expression. In neurons, the apoptotic process was identified through a CASP2-dependent pathway, and a CASP9-dependent pathway was suppressed by endogenous inhibitors of apoptosis proteins [20]. APAF-1 is one of the key molecules in the intrinsic pathway of apoptosis and plays an important role in the formation of the apoptosome complex and caspase activation [21]. Our findings demonstrate that HSP90 inhibition with XL-888 activates the CASP2-mediated apoptosis pathway in these cells. However, it is difficult to conclude that Debio0932 induces the apoptotic pathway in SH-SY5Y cells. Numerous studies have indicated that HSP90 inhibitors reduce cell proliferation by causing apoptosis in cancer cells [5, 6]. Kim et al. revealed that the HSP90 inhibitor geldanamycin reduced cell viability in the SH-SY5Y human neuroblastoma cell line and induced the apoptotic pathway via caspase activation, mitochondrial release of cytochrome c, and subsequent PARP cleavage [22].

The expressions of cell cycle-related genes AURKA, CCND3, CDC20, E2F4, MCM2 and STMN1 changed with XL-888 administration. Accordingly, XL-888 resulted in an increase in AURKA, CCND3, E2F4, and MCM2 gene expressions, while causing a decrease in CDC20 and STMN1 gene expressions. Debio0932 elicited no change in the expression of cell cycle-related genes found in the PCR pathway assay. E2F4 is a transcription factor that contributes to the control of the cell cycle in the G0/G1 phase [23]. MCM2 is a vital regulator of DNA replication and [24] silencing MCM2 triggered G1/S arrest in non-small cell lung carcinoma [25]. CCND3 belongs to the family of D-Cyclin proteins and contributes to G1/S transition control [26]. AURKA is a member of the serine/threonine kinase family, which is considered important in controlling cell cycle and division [27]. CDC20 is a well-known regulator of the cell cycle, as it ensures the completion of cell division by controlling the correct segregation of chromosomes during the mitotic phase (M) [28]. STMN1 expression is increased in some malignancies, and inhibition of its expression may alter the division of tumor cells and thereby arrest cell growth in G2/M phase [29]. Our findings demonstrate that inhibiting the HSP90 protein with XL-888 results in a reduction in the expression of CDC20 and STMN1 genes, leading to cell cycle arrest in the G2/M phase in these cells. Depending on the individual characteristics of the tumor cell, cell cycle arrests have been reported to occur in both G0/1 and G2/M phases following HSP90 inhibition [30]. Wang et al. demonstrated that the HSP90 inhibitor SNX-2112 could inhibit cell growth by inducing apoptosis and G2/M cell cycle arrest in MCF-7 human breast cancer cells [31]. In another study, Hsp90 inhibitors, geldanamycin and 17-allylaminogeldanamycin (17-AAG), induced G2/M arrest along with decreased protein levels of CDC25C and CDC2 in lung cancer cell lines [32].

The expressions of genes associated with cellular senescence, BMI1, ETS2, IGFBP7, MAP2K3, SOD1, changed after XL-888 treatment. Our findings showed that there was a decrease in the expression of BMI, IGFBP7, MAP2K3 and SOD1 genes and an increase in ETS2 gene expression in SH-SY5Y cells. Debio0932 did not induce alterations in the expression of genes associated with cellular senescence. Lee et al. showed that 17-DMAG, an Hsp90 inhibitor, could suppress self-renewal of breast cancer stem cells by downregulating BMI1 expression [33]. Similarly, HSP90 inhibition with XL-888 downregulated BMI1 expression in neuroblastoma cells. IGFBP7 promotes tumor cell proliferation, angiogenesis and epithelial-mesenchymal transition [34]. MAP2K3 has been described as an oncogene whose elimination decreases tumor development and increases biological responsiveness to chemotherapy [35]. SOD1 is an important antioxidant with oncogenic effects in many cancers and is overexpressed in various cancers. [36]. The fact that BMI1, ETS2, IGFBP7, MAP2K3, SOD1 expressions are downregulated in SH-SY5Y cells after XL-888 treatment supports the cytotoxic effect of XL-888 against these cells. ETS2 is a transcription factor that controls gene expression by binding to many genes. It performs a wide range of functions in the cell, including differentiation, proliferation, migration and apoptosis [37]. Wolvetang et al. demonstrated that overexpression of ETS2 induces p53-dependent apoptosis [38]. Our findings showed that XL-888 contributed to the induction of apoptosis in cells by increasing ETS2 expression.

The treatment of XL-888 resulted in changes in the expression of genes associated with DNA damage and repair pathways, including DDIT3, ERCC3, ERCC5, GADD45G, LIG4, and PPP1R15A. Specifically, XL-888 led to a decrease in DDIT3, ERCC5, and PPP1R15A gene expressions, while ERCC3 expression increased in SH-SY5Y cells. On the other hand, the administration of Debio0932 in these cells decreased GADD45G expression while increasing LIG4 expression. Lin et al. revealed that DDIT3 is associated with poor prognosis and overexpression of DDIT3 increases cell proliferation and colony-forming ability in gastric cancer [39]. The ERCC3 and ERCC5 gene is a central component of the nucleotide excision repair pathway [40]. PPP1R15A is another gene involved in DNA damage and repair mechanisms and has been reported to be overexpressed in association with chemoresistance in acute myeloid leukemia [41]. GADD45G has been shown to be involved in the regulation of many cellular functions including genotoxic stress cell cycle control, DNA repair and aging [42]. We showed that XL-888 and Debio0932 down-regulated DNA repair-related genes DDIT3, ERCC5, PPP1R15A and GADD45G to inhibit neuroblastoma cell proliferation. However, unexpectedly, HSP90 inhibition caused an increase in the expression of DNA damage repair genes ERCC3 and LIG4. However, the dual role of the LIG4 enzyme in cancer has been mentioned in the literature. According to several research, large levels of this enzyme give a favorable response to DNA damage repair in cancer, preserving genomic stability. However, increased LIG4 enzyme levels may result in a worse treatment response due to its better ability to repair damage induced by chemotherapy or radiation [43]. There are findings in previous studies that HSP90 inhibition has a direct effect on DNA repair mechanisms. McLaughlin et al. revealed that HSP90 inhibition with the HSP90 inhibitor AUY922 sensitizes head and neck cancer to platinum-based chemotherapy and radiotherapy by modulating the DNA damage response [8]. Our findings indicate that XL-888 and Debi0932 HSP90 inhibitors are important candidates for sensitizing neuroblastoma cells to radiotherapy and chemotherapy.

HSP90 inhibition led to changes in the expression of epithelial to mesenchymal transition (EMT)-related genes CDH2, DSP, FOXC2, GSC, SNAI2 and SOX10. Accordingly, while XL-888 increased CDH2 and DSP gene expressions, it decreased SNAI2 expression. Debio0932 caused a decrease in FOXC2, GSC, SANI2 and SOX10 expressions. Lammens et al. have shown in their study that low CDH2 expression is strongly associated with metastasis in neuroblastoma [44]. DSP proteins provide the close connection between desmosomal cadherins and the cytoskeleton by interacting with γ-catenin and intermediate filaments. Many studies have revealed that desmosome decrease is related to invasive aggressiveness in tumor cells [45]. In our study, XL-888 caused an increase in both CDH2 and DSP expression levels in neuroblastoma cells. This demonstrated the potential of XL-888 in inhibiting invasion of neuroblastoma cell lines. SNAI2 is an EMT-transcription factor that plays an important role in neural crest cell migration and survival. Vrenken et al. revealed that loss of SNAI2 function in neuroblastoma cells strongly sensitizes these cells to retinoic acid therapy while reducing self-renewal, invasion, and metastatic spread in vivo [46]. FOXC2, GSC, and SOX10 are transcription factors that are upregulated in various types of cancer and have been associated with invasion and metastasis [47–49]. Our findings indicate that XL-888 and Debio0932 may block the EMT process by regulating the expression of EMT-related genes in neuroblastoma cells.

HSP90 inhibition also affected the expression of hypoxia signaling-related genes. Interestingly, XL-888 treatment led to an upregulation in the expression of hypoxia signaling-related genes HMOX1 and SLC2A1, whereas Debio0932 treatment resulted in a downregulation of CA9 and HMOX1 expression. Overexpression of HMOX1 has been identified in a variety of cancers and is linked to immune evasion, angiogenesis, cancer cell proliferation, invasion, and treatment resistance [50]. SLC2A1 encodes GLUT1, a glucose transporter involved in the metabolism of glucose, which provides an energy source for cell proliferation and contributes to cancer progression and development [51]. CA9 is a tumor-associated transcription factor that is inducible in hypoxia. In many adult-type epithelial, brain cancers and neuroblastoma cells, increased CA9 expression has been linked to a poor prognosis [52]. Our findings showed that Debio0932 may be effective in SH-SY5Y cells by reducing the expression of hypoxia signaling genes in these cells, while XL-888 was not effective in downregulating hypoxia signaling genes. These results indicate that HSP90 inhibitors may trigger different cellular responses by selectively targeting specific signaling pathways, thus potentially leading to different therapeutic strategies in cancer treatment.

Expression levels of metabolism-related genes ACLY, ATP5A1 COX5A, CPT2, G6PD, LPL, PFKL also changed following treatment with HSP90 inhibitors. XL-888 resulted in upregulation of ACLY, CPT2, G6PD, and PFKL genes, while downregulating expressions of ATP5A1 and COX5A genes. Similarly, Debio0932 led to a reduction in LPL gene expression. Genes such as ACLY, CPT2, G6PD LPL and PFKL are part of metabolic pathways such as lipid metabolism and energy production [53–55]**.** Therefore, XL-888 increasing the expression of these genes may result in acceleration or enhancement of these metabolic processes. On the other hand, genes such as ATP5A1 and COX5A are associated with cellular respiration and ATP synthesis [56]. Downregulating the expression of these genes can lead to a decrease in cellular energy production. Consequently, this could result in reduced metabolic activity within cancer cells, thereby leading to a decrease in cell proliferation. In addition, HSP90 inhibition with XL-888 increased TNKS2 gene expression and decreased DKC1 gene expression which are telomere and telomerase-related genes, Debio0932 did not induce any alterations in these genes. TNKS2 is part of a protein complex involved in the length regulation of telomerase and may increase telomerase activity. Therefore, the increase in TNKS2 gene expression may promote the proliferation of cancer cells [57]. DKC1 plays an important role in the biosynthesis of telomerase and contributes to the creation of a stable telomerase enzyme. Wang et al. observed that DKC1 gene deletion drastically decreased neuroblastoma cell activity, migration, invasion, and proliferation [58]. Villa et al. showed that the HSP90 inhibitors 17-AAG and Geldanamycin caused a significant inhibition of telomerase activity in JR8 human melanoma cells [59]. Intracellular biochemical networks often consist of many interacting components. Increasing or decreasing the expression of one protein can indirectly affect the expression of other proteins. Therefore, the fact that XL-888 affects metabolism and telomerase in two different directions in their expression may be the result of a series of interactions occurring in complex regulatory networks.

**Fig. 3:**
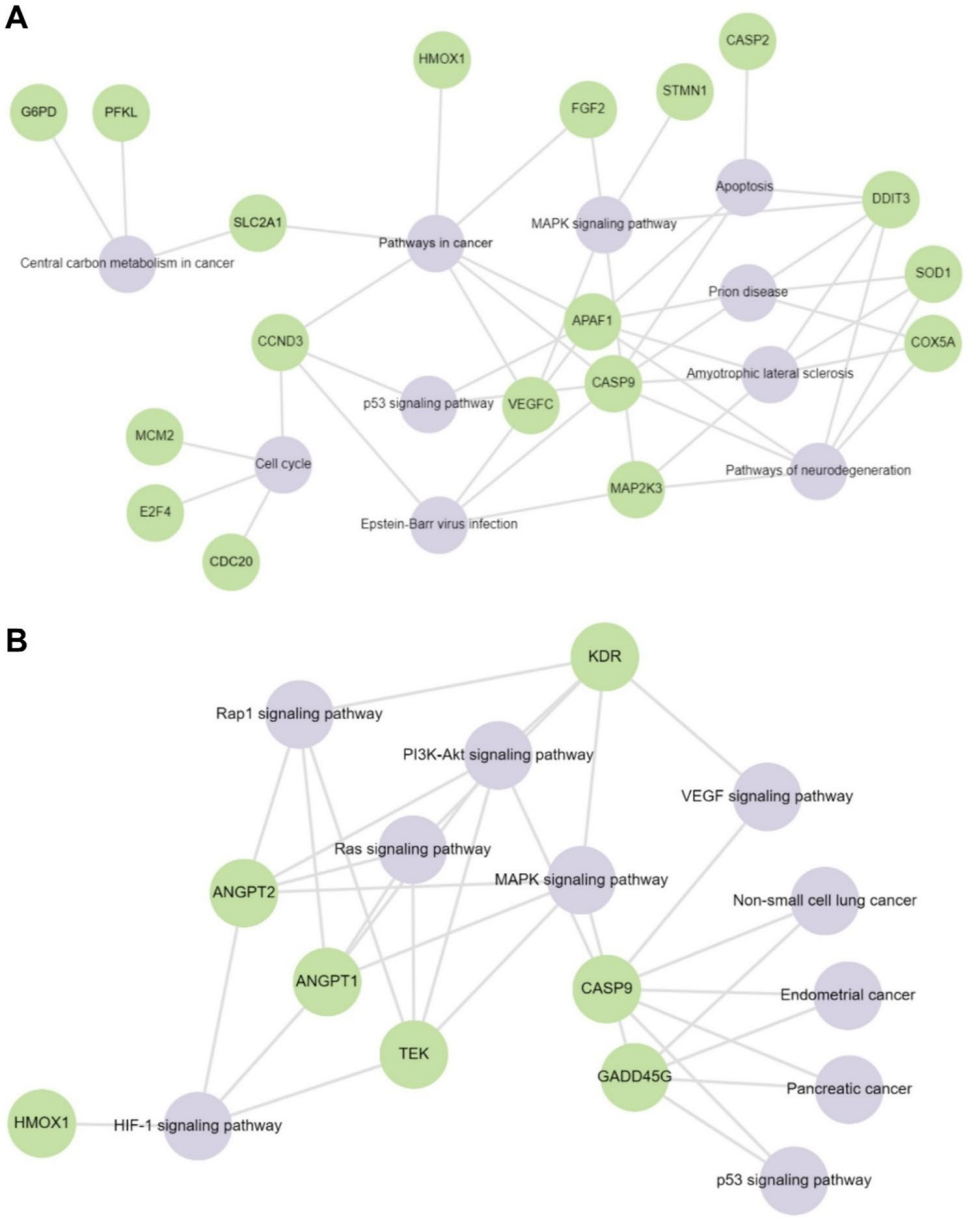
A network represented by pathway from the KEGG database consisting of significantly enriched terms in SH-SY5Y cells treated with XL-888 (**A**) and Debio0932 (**B**)

The Enrichr online tool was used to analyze pathways including both downregulated and upregulated genes. The qRT-PCR analysis experiment revealed 34 significant down- and up-regulated genes in SH-SY5Y cells as a result of XL-888 treatment. Under the effect of Debio0932, 14 down- and up-regulated genes were identified. The relationship of these genes with the signaling pathway was determined using the KEGG pathway (Table 2 and Fig. 3).

**Table 2:** The significantly enriched terms in SH-SY5Y cells treated with XL-888 and Debio0932 from the KEGG human gene set library

Drug: XL-888			
Term	*p* value	*q* value	Overlap genes
Pathways in cancer	2.57969E− 05	0.001646243	*CASP9, CCND3, APAF1, SLC2A1, VEGFC, HMOX1, FGF2*
Amyotrophic lateral sclerosis	3.0486E− 05	0.001646243	*MAP2K3, CASP9, APAF1, DDIT3, COX5A, SOD1*
Cell cycle	5.65198E− 05	0.002034714	*CDC20, CCND3, E2F4, MCM2*
Prion disease	9.19213E− 05	0.002050677	*CASP9, APAF1, DDIT3, COX5A, SOD1*
Apoptosis	9.57204E− 05	0.002050677	*CASP9, APAF1, DDIT3, CASP2*
MAPK signaling pathway	0.000130149	0.002050677	*MAP2K3, DDIT3, STMN1, VEGFC, FGF2*
Pathways of neurodegeneration	0.000132914	0.002050677	*MAP2K3, CASP9, APAF1, DDIT3, COX5A, SOD1*
Central carbon metabolism in cancer	0.0002273	0.003068552	*G6PD, PFKL, SLC2A1*
p53 signaling pathway	0.000257363	0.003088355	*CASP9, CCND3, APAF1*
Epstein-Barr virus infection	0.000369444	0.003695745	*MAP2K3, CASP9, CCND3, APAF1*

Drug: Debio0932			
Term	*p* value	*q* value	Genes
HIF-1 signaling pathway	8.01072E− 07	3.93178E− 05	*ANGPT2, ANGPT1, HMOX1, TEK*
MAPK signaling pathway	1.19145E− 06	3.93178E− 05	*ANGPT2, ANGPT1, KDR, TEK, GADD45G*
PI3K-Akt signaling pathway	2.96491E− 06	6.52279E− 05	*CASP9, ANGPT2, ANGPT1, KDR, TEK*
Rap1 signaling pathway	1.08874E− 05	0.000179642	*ANGPT2, ANGPT1, KDR, TEK*
Ras signaling pathway	1.61193E− 05	0.000212775	*ANGPT2, ANGPT1, KDR, TEK*
Endometrial cancer	0.000735474	0.007174915	*CASP9, GADD45G*
VEGF signaling pathway	0.000760976	0.007174915	*CASP9, KDR*
Non-small cell lung cancer	0.001130904	0.008309204	*CASP9, GADD45G*
p53 signaling pathway	0.001162296	0.008309204	*CASP9, GADD45G*
Pancreatic cancer	0.00125897	0.008309204	*CASP9, GADD45G*

**Fig. 4:**
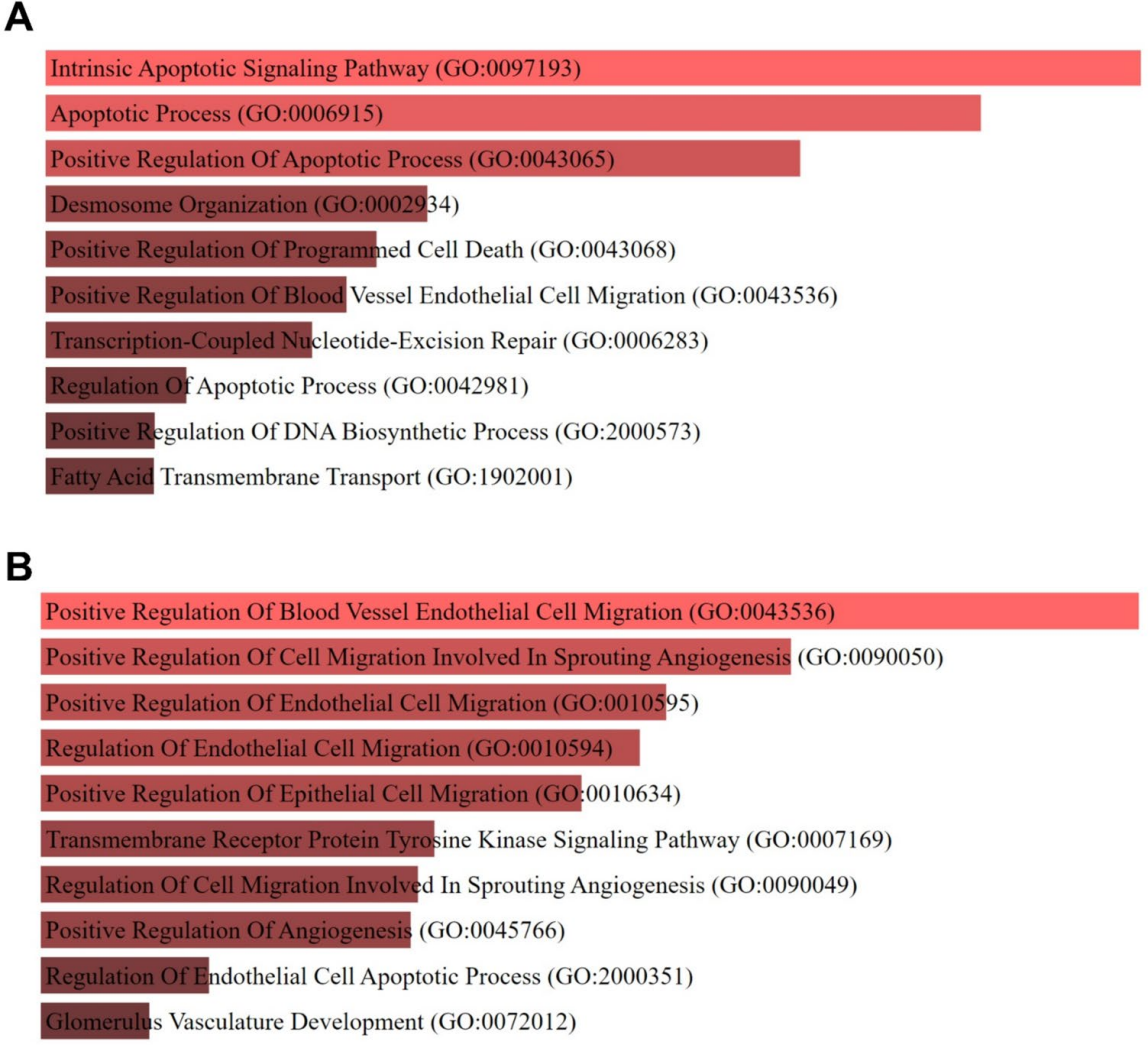
GO Biological Process enrichment analysis A. Top ten GO terms for XL-888 (**A**) and Debio0932 (**B**)

The results revealed that SH-SY5Y cells exposed to XL-888 were closely associated with the signaling pathways of Pathways in cancer, Amyotrophic lateral sclerosis, Cell cycle, Prion disease, Apoptosis, MAPK signaling pathway, Pathways of neurodegeneration, Central carbon metabolism in cancer, p53 signaling pathway, and Epstein-Barr virus infection. In contrast, cells exposed to Debio0932 were linked with the signaling pathways of HIF-1 signaling pathway, MAPK signaling pathway, PI3K-Akt signaling pathway, Rap1 signaling pathway, Ras signaling pathway, Endometrial cancer, VEGF signaling pathway, non-small cell lung cancer, p53 signaling pathway, and Pancreatic cancer. Additionally, the roles of XL-888 and Debio0932 in biological processes were elucidated using Gene Ontology (GO) analysis based on the expression changes of genes (Fig. 4).

The results indicate that HSP90 inhibition by XL-888 and Debio0932 plays a role in the regulation of genes in multiple pathways in neuroblastoma. GO analysis revealed that XL-888 has prominent gene regulations related to apoptosis, angiogenesis, and metastasis, while Debio0932 predominantly regulates biological processes associated with angiogenesis.

**Fig. 5:**
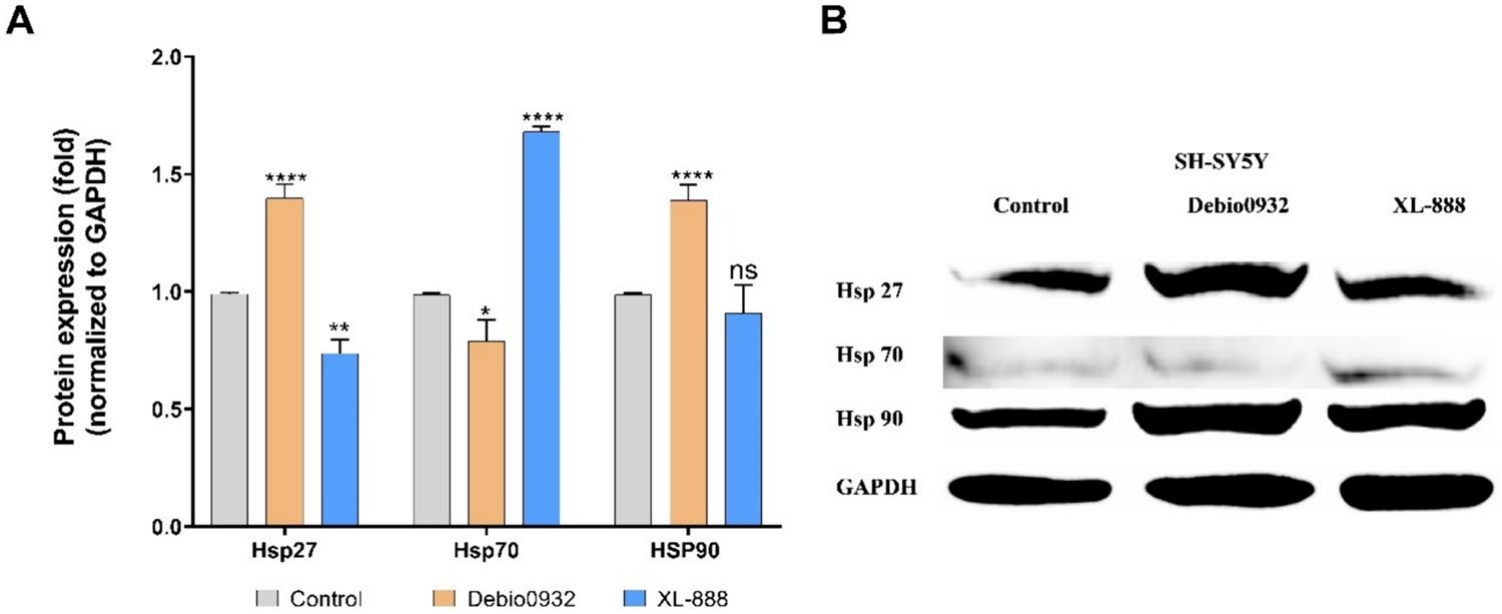
Changes in the expression of heat shock proteins (Hsp27, HSP70, HSP90) in SH-SY-5Y cells after a 48 h treatment with XL-888 and Debio0932. The protein expression (**A**) changes in HSPs (HSP27, HSP70, HSP90) and the abundance HSPs in SH-SY5Y (**B**). (*p < 0.05, **p < 0.01, ***p < 0.001, and ****p < 0.0001, *ns* not significant)

Finally, western blotting analysis was performed to investigate the effect of HSP90 inhibition on the protein abundance of HSPs in SH-SY5Y cells with XL-888 and Debio0932. The western blotting analysis unveiled alterations in the protein expressions of key HSPs (HSP27, HSP70, and HSP90) in cancer. (Fig. 5). XL-888 led to a reduction in HSP27 protein abundance, an elevation in Hsp70 expression, and did not induce a significant alteration in HSP90 protein levels. Similarly, in our previous study, XL-888 significantly increased HSP70 levels in the liver cancer cell lines HUH-7 and HepG2 [5]. The phenomenon is attributable to the separation of the heat shock factor-1 (HSF1) monomer from HSP90, which is followed by HSF1 trimerization, nuclear translocation, and HSP70 transcription activation. In the same study, HSP27 and HSP90 gene levels showed no significant change in HepG2, but a significant decrease in HUH-7 cells. This study’s findings support the idea that each cell line responds differently to XL-888 therapy. HSP27 is known to perform a cytoprotective effect by directly suppressing cell death via interactions with key proteins in the apoptotic cascade [60]. In addition, HSP27 may improve cell survival by regulating the phosphorylation of the ERK and Akt signaling pathways and promoting the degradation of apoptotic components. Upregulation of HSP27 in neuroblastoma cells has been reported in previous studies to inhibit cell apoptosis [61]. In qRT-PCR analysis, the effect of XL-888 on the apoptosis-related genes APAF1, CASP2, CASP9 supports these findings. Debio0932 triggered a significant increase in HSP27 and HSP90 protein expression, while inducing a reduction in Hsp70 protein levels. It has been demonstrated that treatment with Hsp90 inhibitors in cells causes the release of heat shock factor-1 (HSF-1) and overexpression of other HSPs such as Hsp27 and Hsp70, which can result in resistance to Hsp90 inhibitor therapy [62]. HSP90 inhibitors Geldanamycin or 17DMAG have been reported to induce Hsp27 and Hsp70 expression [63].

While HSP90 inhibitors may play a role in regulating different pathways in each cell line, different HSP90 inhibitors may also play a role in regulating different pathways in the same cell line. This is a result of the complex molecular pathways of cancer cells. XL-888 was more effective at lower concentrations in regulating cancer-related pathways in SH-SY5Y cells compared to Debio0932. qRT-PCR and western blotting analyzes show that XL-888 and Debio0932 can be used as a promising agent in therapy in neuroblastoma cell line SH-SY5Y and are effective in many cancer-related pathways. These inhibitors can be effective in the treatment of neuroblastoma using them alone or in combination with other therapeutic agents. Luyao et al. demonstrated that the HSP90 inhibitor AUY-922 was effective in reducing resistance to ALK inhibitors in human neuroblastoma [7]. Combining chemotherapy with HSP90 inhibitors and targeted drugs has the potential to efficiently prevent various carcinogenic signaling pathways while also increasing the anticancer benefits of targeted drugs. Thus, targeting HSP90 is a potential and successful technique for combating cancer resistance.

## Conclusion

In this study, the effect of XL-888 and Debio0932 HSP90 inhibitor on SH-SY5Y neuroblastoma cells was comprehensively evaluated. The results of this study revealed that HSP90 inhibitors XL-888 and Debio0932 exhibited significant anticancer activity in neuroblastoma cells. These inhibitors were found to regulate the expression of many cancer-related genes in neuroblastoma and contribute to the regulation of many cancer-related pathways such as migration, invasion, metastasis, angiogenesis, and apoptosis. These findings demonstrated the importance of using HSP90 inhibitors alone and in combination with other therapy strategies in the treatment of neuroblastoma and other types of cancer.

## Data Availability

Data and materials are available from the authors upon request.
